# Early Antiandrogen Therapy With Dutasteride Reduces Viral Shedding, Inflammatory Responses, and Time-to-Remission in Males With COVID-19: A Randomized, Double-Blind, Placebo-Controlled Interventional Trial (EAT-DUTA AndroCoV Trial – Biochemical)

**DOI:** 10.7759/cureus.13047

**Published:** 2021-02-01

**Authors:** Flavio A Cadegiani, John McCoy, Carlos Gustavo Wambier, Andy Goren

**Affiliations:** 1 Internal Medicine: Diabetes and Endocrinology, Applied Biology Inc, Irvine, USA; 2 Clinical Endocrinology, Federal University of São Paulo, São Paulo, BRA; 3 Dermatology, Research and Development, Applied Biology Inc, Irvine, USA; 4 Dermatology, Warren Alpert Medical School of Brown University, Providence, USA; 5 Dermatology, Applied Biology Inc, Irvine, USA

**Keywords:** covid-19, sars-cov-2, dutasteride, nitazoxanide, azithromycin

## Abstract

Background and objective

Severe acute respiratory syndrome coronavirus 2 (SARS-CoV-2) cell entry and subsequent infectivity are mediated by androgens and the androgen receptors through the regulation of transmembrane protease, serine 2 (TMPRSS2). Androgenetic alopecia (AGA) predisposes males to severe coronavirus disease 2019 (COVID-19) disease, while the use of 5-alpha-reductase inhibitors (5ARis) and androgen receptor antagonists reduce COVID-19 disease severity. In this study, we aimed to determine the potential benefit of dutasteride, a commonly used broad and potent 5ARi, as a treatment for COVID-19.

Design, setting, and participants

The study was conducted at outpatient clinics. Subjects presented to the clinics with a positive reverse transcription-polymerase chain reaction (RT-PCR) test taken within 24 hours of recruitment. All subjects presented with mild to moderate symptoms.

Interventions

Subjects were given either dutasteride 0.5 mg/day or placebo for 30 days or until full COVID-19 remission. All subjects received standard therapy with nitazoxanide 500 mg twice a day for six days and azithromycin 500 mg/day for five days.

Main outcome(s) and measure(s)

The main outcome(s) and measure(s) were as follows: time to remission, oxygen saturation (%), positivity rates of RT-PCR-SARS-CoV-2, and biochemical analysis [ultrasensitive C-reactive protein (usCRP), D-dimer, lactate, lactate dehydrogenase (LDH), erythrocyte sedimentation rate (ESR), ultrasensitive troponin, and ferritin].

Results

Subjects taking dutasteride (n=43) demonstrated reduced fatigue, anosmia, and overall disease duration compared to subjects taking a placebo (n=44) (p<.0001 for all). Compared to the placebo group, on Day seven, subjects taking dutasteride had a higher virologic remission rate (64.3% versus 11.8%; p=.0094), higher clinical recovery rate (84.7% versus 57.5%; p=.03), higher mean [standard deviation: SD] oxygen saturation (97.0% [1.4%] versus 95.7% [2.0%]; p=.02), lower median [Interquartile range: IQR] usCRP (0.34 mg/L [0.23 mg/L-0.66 mg/L] versus 1.47 mg/L [0.70 mg/L-3.37 mg/L]; p<.0001), lower median [IQR] lactate (2.01 mmol/L [1.12 mmol/L-2.43 mmol/L] versus 2.66 mmol/L [2.05 mmol/L-3.55 mmol/L]; p=.0049), lower median [IQR] ESR (5.0 mm/1h [3.0 mm/1h-11.0 mm/1h] versus 14.0 mm/1h [7.25 mm/1h-18.5 mm/1h]; p=.0007), lower median [IQR] LDH (165 U/L [144 U/L-198 U/L] versus 210 U/L [179 U/L-249 U/L]; p=.0013) and lower median [IQR] troponin levels (0.005 ng/mL [0.003 ng/mL-0.009 ng/mL] versus 0.007 ng/mL [0.006 ng/mL-0.010 ng/mL]; p=.048).

Conclusions and relevance

The findings from this study suggest that in males with mild COVID-19 symptoms undergoing early therapy with nitazoxanide and azithromycin, treatment with dutasteride reduces viral shedding and inflammatory markers compared to males treated with a placebo.

## Introduction

Coronavirus disease 2019 (COVID-19) disease burden disproportionately falls on men compared to women [1,2], which is not fully explained by sex disparities in terms of lifestyle and comorbidities. We have previously reported that androgen-mediated phenotype of androgenetic alopecia (AGA) in males and hyperandrogenism in females are associated with COVID-19 disease severity [3-5], while the use of antiandrogens is associated with a lower disease burden [6-8].

Severe acute respiratory syndrome coronavirus 2 (SARS-CoV-2) entry into cells is dependent on a cleavage of the viral spike protein by the transmembrane protease, serine 2 (TMPRSS2) expressed on the surface of human cells. The only known promoter of the TMPRSS2 enzyme is an androgen response element located in the 5’ promoter region [9,10]. As such, it is plausible to hypothesize that SARS-CoV-2 viral infectivity is regulated by androgens, as explained in several communications that we have published suggesting that the male bias in COVID-19 disease severity may be linked to androgens, and reinforced by the disease patterns according to the androgenic phenotypes in both males and females [3-5, 11-13]. Accordingly, the reduction of the TMPRSS2 expression by blocking the androgen receptor would decrease SARS-CoV-2 entry into human cells [6-8, 13-17], which is corroborated by studies showing protection from more severe states related to COVID-19 with the use of antiandrogens [6-8]. In addition, variation in the androgen receptor gene may predict COVID-19 disease severity [13,18]. Taken together, there is sufficient evidence to explore more about the use of drugs that reduce androgen receptor as a promising therapeutic option against COVID-19.

5-alpha-reductase inhibitors (5ARis) are commonly prescribed antiandrogens for AGA and benign prostatic hyperplasia (BPH). Their mechanism of action involves the blockage of the conversion of testosterone to dihydrotestosterone (DHT), a more potent androgen [11]. 5ARis are inexpensive and have a relatively low incidence of adverse side effects. Because of the mechanistic plausibility and increasing evidence of the role of antiandrogens as protective agents against COVID-19, we conducted the Early Antiandrogen Treatment With Dutasteride for COVID-19 (EAT-DUTA AndroCoV) Trial, a double-blinded, placebo-controlled randomized clinical trial (RCT), which aimed to assess the efficacy of early antiandrogen therapy (EAT) with the use of dutasteride (DUTA) as a treatment for COVID-19. The present study is an analysis of the biochemical, virological, and clinical profile of a subset of participants of the EAT-DUTA AndroCoV Trial who randomly underwent a more comprehensive biochemical assessment, as determined previously at the beginning of the RCT.

## Materials and methods

Study design 

Potential subjects were recruited for a double-blinded, randomized, prospective, investigational study of antiandrogen treatment of COVID-19 through social media, a patient mailing list containing 10,900 men from a Brasilia-based Brazilian healthcare system registry, as well as referrals from other physicians. For the present study, subjects presented to an outpatient clinic with a confirmed positive reverse transcription-polymerase chain reaction test for SARS-CoV-2 (RT-PCR-SARS-CoV-2). The study was registered and approved by the Brazilian National Ethics Committee [approval number: 4.173.074; process number (CAAE): 34110420.2.0000.0008; Comitê de Ética em Pesquisa (CEP), Conselho Nacional de Ética em Pesquisa (CONEP), Ministry of Health (Ministério da Saúde - (MS) (CEP/CONEP/MS)]. All patients admitted to the study signed informed consent. Baseline characteristics, presence of comorbidities, use of medications, clinical characteristics of COVID-19, test results, and disease outcomes were recorded by the principal investigator and managed by the study director.

Study population

Screening of subjects suspected for COVID-19 was conducted by the principal investigator at the major site of the research (Corpometria Institute Brasilia, Brazil). SARS-CoV-2 status was laboratory-confirmed by the RT-PCR-SARS-CoV-2 kit testing (Automatized Platform, Roche USA, Indianapolis, IN) following the COBAS SARS-CoV-2 RT-PCR kit test protocol. Additional inclusion criteria were as follows: (1) males older than 18 years of age; (2) COVID-19 infection lasting less than seven days; (3) absence of specific treatments for COVID-19 for periods longer than 72 hours; (4) no prior adverse reaction to dutasteride; (5) oxygen saturation (SaO_2_) above 92% at the time of enrollment; and (6) no signs of complications related to COVID-19 and secondary bacterial infections at the time of screening.

Procedures

Patients were randomized for either dutasteride or placebo groups. Dutasteride was given at a dose of 0.5 mg/day for 30 days or until full COVID-19 remission. For all subjects, nitazoxanide 500 mg twice a day was given after meals for six days and azithromycin 500 mg/day was given one hour before meals for five days, as per the protocol of one of the standardized therapies suggested by the local Ministry of Health.

All participants enrolled in the study were evaluated for clinical outcomes, and a subset of two-thirds of the subjects underwent biochemical analysis in a random manner due to budget limitations. The biochemical/virological analysis aimed to detect further differences in inflammatory and virological responses between the dutasteride and placebo groups. A 2:1 randomization was performed during enrollment for biochemical evaluation on at least two time points between Day zero, Day seven, and Day 14, and patients were selected for the present analysis. Clinical outcomes were adjusted for the subset of patients that were selected for the present study.

Study outcomes

Endpoints for the study were remission times for fatigue and loss of taste or smell (ageusia or anosmia), time to overall COVID-19 remission including and not including anosmia and ageusia. Time to overall COVID-19 remission, or COVID-19 ‘cure’, was defined as full remission of symptoms. Disease recovery was evaluated through a report of perceived recovery scale from 0 to 100: 100 being completely healthy (fully recovered) and 0 being their worst day of COVID-19; subjects rated their recovery on Day zero (start of treatment), and Day one, Day two, Day three, Day seven, Day 14, Day 21, and Day 30. The percentage of patients completely free of clinical symptoms of COVID-19 was determined on Day seven. Oxygen saturation (%) and heart rate (bpm) were measured on Day zero, Day one, Day two, Day three, and Day seven.

Biochemical parameters of the subset of subjects enrolled in the EAT-DUTA AndroCoV trial included RT-PCR-SARS-CoV-2 on Day seven, ultrasensitive C-reactive protein (usCRP, mg/L) (latex-intensified immunoturbidimetry), D-dimer (ng/mL) (immunologic assay), lactate (mmol/L) (enzymatic assay), lactate dehydrogenase (LDH, U/L) (lactate NAD), erythrocyte sedimentation rate (ESR, mm/1h) (capillary photometry), ultrasensitive troponin (ng/mL) (electric chemiluminescence - ECLIA) and ferritin (ng/mL) (chemiluminescence - CLIA), in pre-COVID (if available), and on Day zero, Day seven, Day 14, delta Day seven to zero, delta Day 14-0, and percentage of different levels of reductions between Days zero and seven and between Days zero and 14.

Additional records included neutrophils (*1000/mm^3^), lymphocytes (*1000/mm^3^), eosinophils (*1000/mm^3^), and monocytes (*1000/mm3) (flow cytometry with fluorescent protein - XN10-Sysmex); and the neutrophil-to-lymphocyte ratio was then calculated. 

Sample size calculations

Based on the assumptions that sample size should be estimated for the chi-squared test to detect the difference in proportions at p=0.05 for inflammatory markers in COVID-19, that the number of subjects enrolled in arm A is equal to the number of subjects in arm B, i.e., 1:1 enrollment ratio, and that 95% of subjects will complete the study, we calculated that we needed to recruit a minimum number of 70 subjects.

Statistical analysis

Medical history, concomitant medications, and lifestyle characteristics of COVID-19 patients were tabulated based on the following characteristics for each group: age, BMI, hypertension, myocardial infarction (MI), stroke, heart failure, lipid disorders, diabetes, prediabetes, obesity, asthma, chronic obstructive pulmonary disease (COPD), cancer, benign prostatic hyperplasia, prostate cancer, chronic renal disease, liver fibrosis/cirrhosis, clinical depression, anxiety, attention deficit hyperactivity disorder (ADHD), insomnia, hypogonadism, hypothyroidism, and autoimmune disorders as well as indicated medications. Mean and standard deviations (SD) were used for clinical parameters and median and interquartile range (IQR) were presented for biochemical parameters. Non-parametric statistical tools were employed to determine statistical significance, which was set at p<0.05. XLSTAT version 2020.3.1.1008 (Addinsoft, Inc. New York, NY) was used to perform all statistical analyses.

## Results

**Figure 1:**
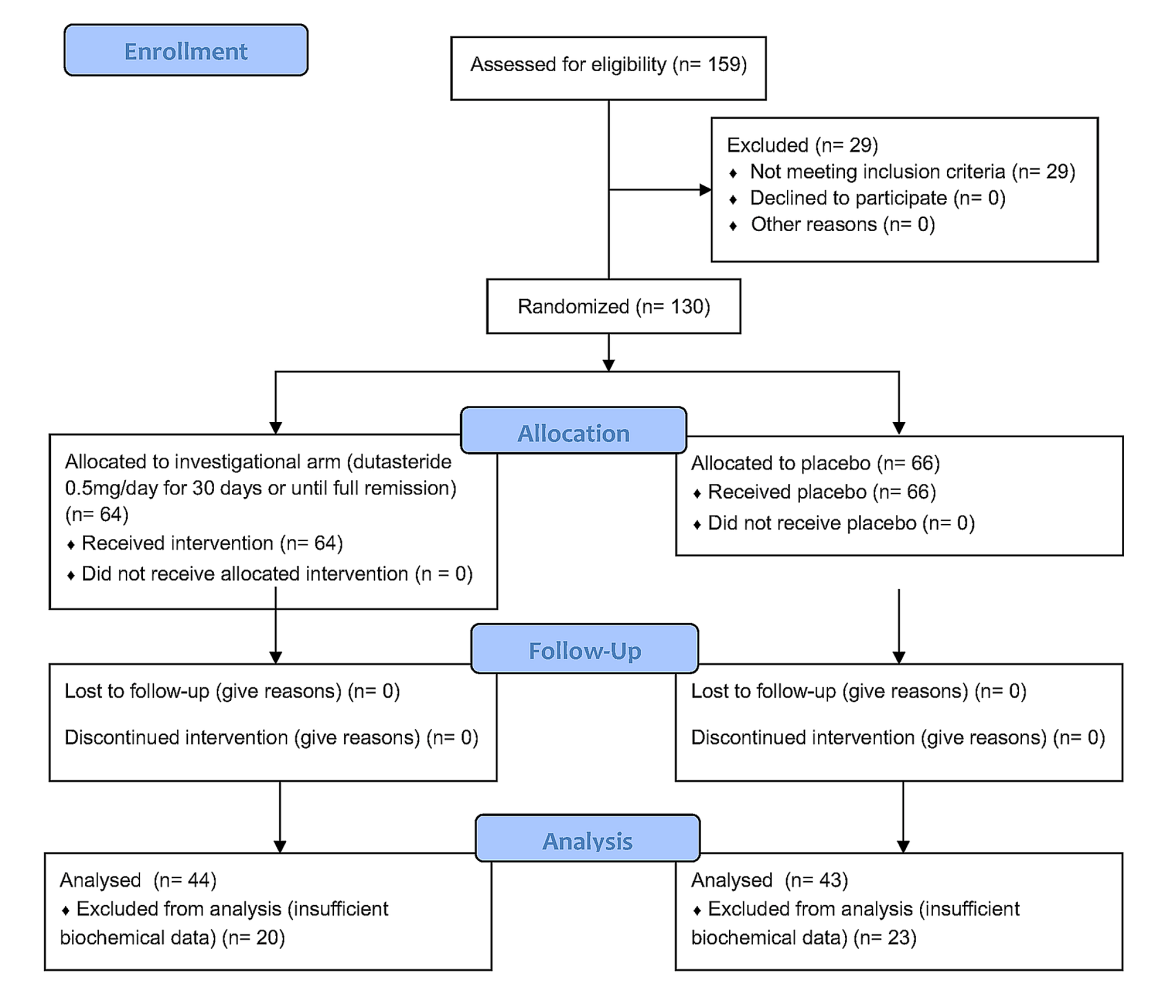
CONSORT flow diagram for EAT-DUTA AndroCoV-Trial biochemical arm CONSORT: Consolidated Standards of Reporting Trials; EAT-DUTA AndroCoV: Early Antiandrogen Treatment With Dutasteride for COVID-19

Of the 138 participants enrolled in the EAT-DUTA AndroCoV Trial, a subset of 87 subjects underwent biochemical analyses, including 43 men from the dutasteride group and 44 men from the placebo group (Figure 1).

Baseline characteristics, the prevalence of comorbidities, and use of medications in the two study groups are presented in Table 1; they were found to be similar for all parameters. The average interval between first symptoms and beginning of treatment was 4.3 days for both groups (p=n/s). Both groups received additional therapies for COVID-19 in similar percentages. All 13 participants that used glucocorticoid therapy, including six (13.9%) in the placebo group and seven (15.9%) in the dutasteride group received the same drug (dexamethasone) at a dose of 6 mg/day for a duration of three days.

**Table 1:** Characteristics of the study populations MI: myocardial infarction; CHF: congestive heart failure; T2DM: type 2 diabetes mellitus; T1DM: type 1 diabetes mellitus; COPD: chronic obstructive pulmonary disorder; CKD: chronic kidney disease; ADHD: attention deficit hyperactivity disorder; BPH: benign prostate hyperplasia; ACEi: angiotensin-converting enzyme inhibitors; ARB: angiotensin-2 receptor blockers; CCB: calcium channel blocker; GLP1Ra: glucagon-like peptide-1 receptor analogues; SGLT2i: sodium-glucose cotransporter-2 inhibitors; DPP4i: dipeptidyl-peptidase 4 inhibitors; P: progesterone; E: estradiol; GnRH: gonadotropin release hormone; SERM: selective estrogen receptor modulators; NSAA: non-steroidal antiandrogen; SSRI: selective serotonin reuptake inhibitors; CNS: central nervous system; BCG: Bacillus Calmette-Guérin; SD: standard deviation; n/s: not significant; n/a: not applicable

	Placebo group (n=44)	Dutasteride group (n=43)	P-value
Baseline characteristics, mean (SD)			
Age, years	43.8 (14.1)	40 (10.8)	n/s
BMI, kg/m^2^	26.1 (2.2)	26.0 (3.2)	n/s
Time-to-treat, days	4.4 (1.4)	4.3 (1.4)	n/s
Comorbidities, n (%)			
Obesity	4 (9.3%)	5 (11.4%)	n/s
Hypertension	12 (27.9%)	7 (15.9%)	n/s
MI	0	0	n/a
Stroke	0	0	n/a
CHF	0	1 (2.3%)	n/a
Lipid disorders	9 (20.9%)	9 (20.4%)	n/s
Other cardiac dysfunctions	0	1 (2.3%)	n/a
T1DM and T2DM	4 (9.3%)	4 (9.1%)	n/s
Prediabetes	8 (18.6%)	5 (11.4%)	n/s
Dysglycemia	12 (28.0%)	9 (20.6%)	n/s
Asthma	1 (2.3%)	0	n/a
COPD	0	0	n/a
CKD	0	0	n/a
Liver fibrosis/cirrhosis	1 (2.3%)	0	n/a
Clinical depression	2 (4.6%)	3 (6.8%)	n/s
Anxiety	6 (13.9%)	8 (18.2%)	n/s
ADHD	5 (11.6%)	3 (6.8%)	n/s
Insomnia	4 (9.3%)	1 (2.3%)	n/s
Hypothyroidism	1 (2.3%)	4 (9.1%)	n/a
Autoimmune disorders	1 (2.3%)	1 (2.3%)	n/a
Cancer	0	0	n/a
Erectile dysfunction	2 (4.6%)	1 (2.3%)	n/a
Hypogonadism	8 (18.6%)	8 (18.2%)	n/a
BPH	2 (4.6%)	2 (4.5%)	n/s
Drugs, n (%)			
Beta-blocker	2 (4.6%)	1 (2.3%)	n/a
ECAi	1 (2.3%)	0	n/a
ARB	11 (25.6%)	7 (15.9%)	n/s
Loop diuretics	1 (2.3%)	0	n/a
Thiazide diuretics	4 (9.3%)	1 (2.3%)	n/a
CCB	2 (4.6%)	2 (4.5%)	n/s
Statins	8 (18.6%)	8 (18.2%)	n/s
Others	0	0	n/a
Aspirin	0	1 (2.3%)	n/a
Clopidogrel	0	0	n/a
Warfarin	0	0	n/a
Xa factor inhibitors	0	0	n/a
Direct thrombin inhibitors	0	0	n/a
Heparin	0	0	n/a
Metformin	10 (23.3%)	6 (13.6%)	n/s
GLP1R analogues	2 (4.6%)	3 (6.8%)	n/a
SGLT2 inhibitors	7 (16.3%)	3 (6.8%)	n/s
DPP4 inhibitors	2 (4.6%)	1 (2.3%)	n/a
Sylfonylureas	0	0	n/a
Glitazones	0	0	n/a
Acarbose	0	0	n/a
Insulin	0	0	n/a
Orlistat	2 (4.6%)	2 (4.5%)	n/a
Levothyroxine	1 (2.3%)	4 (9.1%)	n/a
Liothyronine	1 (2.3%)	0	n/a
Testosterone	7 (16.3%)	6 (13.6%)	n/s
Aromatase inhibitors or SERMs	2 (4.6%)	2 (4.5%)	n/s
Hypnotics	3 (7.0%)	1 (2.3%)	n/a
SSRI	3 (7.0%)	6 (13.6%)	n/s
Other antidepressants and humor stabilizers	4 (9.3%)	4 (9.1%)	n/s
Benzodiazepines	1 (2.3%)	0	n/a
Atypical antipsychotics	3 (7.0%)	2 (4.5%)	n/a
CNS stimulants	5 (11.6%)	5 (11.4%)	n/s
Alpha-1 adrenaline blockers	2 (4.6%)	2 (4.5%)	n/s
GnRH analogues and inhibitors, NSAA and others antiandrogens	0	0	n/a
Omega-3	3 (7.0%)	1 (2.3%)	n/a
Vitamin D	13 (30.2%)	15 (34.1%)	n/s
Zinc	6 (13.9%)	7 (15.9%)	n/s
Biotin	1 (2.3%)	0	n/a
Vitamin C	8 (18.6%)	7 (15.9%)	n/s
Multivitamin	1 (2.3%)	2 (4.5%)	n/a
Vaccines and lifestyle, n (%)			
BCG vaccine	43 (100%)	44 (100%)	n/s
Influenza vaccine (in 2020)	14 (32.6%)	14 (31.8%)	n/s
Pneumococcal vaccine (since 2017)	3 (7.0%)	5 (11.4%)	n/s
Current smoking	2 (4.6%)	2 (4.5%)	n/a
Regular physical activity	28 (65.1%)	32 (72.7%)	n/s
Additional COVID-19 treatments, n (%)			
Ivermectin	6 (13.9%)	6 (13.6%)	n/s
Hydroxychloroquine	4 (9.3%)	3 (6.8%)	n/s
Xa factor inhibitors	8 (18.6%)	3 (6.8%)	n/s
Enoxaparin	3 (7.0%)	3 (6.8%)	n/s
Glucocorticoids	6 (13.9%)	7 (15.9%)	n/s
Vitamin C	4 (9.3%)	4 (9.1%)	n/s
Zinc	3 (7.0%)	3 (6.8%)	n/s
Vitamin D	1 (2.3%)	2 (4.5%)	n/a
Colchicine	0	0	n/a
Bromhexine	0	0	n/a
N-acetylcysteine	0	0	n/a

Table 2 illustrates the mean time-to-remission for major clinical symptoms and overall time to remission, patient-reported outcome rating regarding disease severity on Day one, Day two, Day three, and Day seven, and oxygen saturation on Day zero, Day seven, and Day 14 in men taking dutasteride versus men taking placebo.

**Table 2:** Clinical outcomes SD: standard deviation; n/s: not significant

	Placebo group (n=44)	Dutasteride group (n=43)	P-value
Time-to-remission, days, mean (±SD)			
Fatigue	10.3 (±8.4)	5.5 (±3.2)	<0.001
Loss of taste or smell (ageusia or anosmia)	11.1 (±6.6)	5.6 (±4.0)	<0.001
Remission minus taste or smell loss	11.7 (±7.7)	7.0 (±2.9)	<0.001
Overall symptoms	16.3 (±8.3)	9.2 (±4.3)	<0.001
Clinical recovery, mean (±SD)			
% fully clinically recovered on Day 7	57.5%	84.1%	0.03
% of clinical recovery on Day 1	34.2 (±21.4)	60.4 (±24.2)	<0.0001
% of clinical recovery on Day 2	52.9 (±21.3)	78.5 (±17.8)	<0.0001
% of clinical recovery on Day 3	66.8 (±20.7)	89.2 (±12.3)	<0.0001
% of clinical recovery at Day 7	82.9 (±15.0)	97.4 (±5.7)	<0.0001
Oxygen saturation, mean (±SD)			
Day 0	95.4 ±1.4	96.0 ± 1.5	n/s (.21)
Day 7	95.7 ±2.0	97.0 ± 1.4	0.02
Day 14	96.2 ±1.4	97.5 ± 1.2	0.0012
D Day 7-0	+0.3	+1.3	0.047
D Day 14-0	+0.9	+1.3	n/s (.25)

The mean (±SD) remission time for recovery of fatigue was 5.5 (±3.2) days in the dutasteride group versus 10.3 (±8.4) days in the placebo group (p<0.001), representing a 46.6% reduction. The mean remission time for loss of taste or smell was 5.6 (±4.0) days in the dutasteride group versus 11.1 (±6.6) days in the placebo group (p<0.001), signifying a 49.6% reduction. The time to full remission was 9.2 (±4.3) days in the dutasteride group versus 16.3 (±8.3) days in the placebo group (43.2% reduction; p<0.001). When anosmia and ageusia were excluded from the analysis, the average time to full remission was 7.0 (±2.9) days in the dutasteride group versus 11.7 (±7.7) (43.6% reduction; p<0.001).

When compared to baseline (Day zero), the level of recovery was significantly improved in the dutasteride group compared to the placebo group for Day one, Day two, Day three, Day seven (p<0.0001 for all times). The percentage of patients still affected on Day seven was 15.9% in the dutasteride group and 42.5% in the placebo group.

Mean oxygen saturation was higher in the dutasteride group for Day seven (p=0.02) and Day 14 (p=0.0012); so was the level of oxygen saturation increase from baseline (Day zero) to Day seven (p=0.047).

Table 3 presents the virological and biochemical parameters evaluated, including the percentage of subjects with negative RT-PCR-SARS-CoV-2 on Day seven and Day 14; levels of usCRP, lactate, ESR, LDH, ultrasensitive troponin, D-dimer, and ferritin levels on Day zero, Day seven, and Day 14; changes between baseline (Day zero) and Day seven and between baseline (Day zero) and Day 14; and the percentage of men with specific goals for each parameter between baseline (Day zero) and Day seven and between baseline (Day zero) and Day 14.

**Table 3:** Biochemical results IQR: interquartile range; n/s: not significant; n/a: not applicable; RT-PCR: reverse transcription-polymerase chain reaction; usCRP: ultrasensitive C-reactive protein; ESR: erythrocyte sedimentation rate; LDH: lactate dehydrogenase

	Placebo group (n=44)	Dutasteride group (n=43)	P-value
RT-PCR-SARS-CoV-2 remission (CT >40 cycles) (%)			
Day 0	0%	0%	n/s (1.00)
Day 7	11.8%	64.3%	.0094
Day 14	54.2%	88.3%	.036
usCRP (mg/L), median (IQR)			
Pre-COVID	0.18 (0.08-0.41)	0.38 (0.10-0.56)	n/s (.45)
Day 0	1.44 (0.72-2.54)	1.22 (0.83-2.26)	n/s (.42)
Day 7	1.47 (0.70-3.37)	0.34 (0.23-0.66)	<0.0001
Day 14	0.39 (0.21-2.32)	0.36 (0.08-0.34)	0.0026
D Day 7-0	-64.7%	-83.9%	n/a
D Day 14-0	-73.3%	-93.7%	n/a
Lactate (mmol/L), median (95% CI)			
Pre-COVID	0.93 (0.82-1.18)	0.88 (0.72-1.23)	n/s (.58)
Day 0	1.72 (1.35-2.11)	1.51 (1.16-2.01)	n/s (.27)
Day 7	2.66 (2.05-3.55)	2.01 (1.12-2.43)	0.0049
Day 14	1.92 (1.38-2.89)	1.48 (1.22-1.89)	0.014
D Day 7-0	+0.97 (+0.57 - +1.69)	+0.28 (+0.02 - +0.85)	0.025
Lactate increase	19.2%	60%	n/a
D Day 14-0	+0.22 (-0.64 - +0.79)	-0.19 (-1.15 - +0.27)	n/s (.24)
Lactate increase of <0.5 mmol/L between Days 0 and 14	67.8%	83.3%	n/a
ESR (mm/1h), median (IQT)			
Pre-COVID	4.5 (2.25-8.0)	4.0 (2.0-7.0)	n/s (.76)
Day 0	13.5 (7.0-22.5)	13.0 (6.5-22.0)	n/s (.69)
Day 7	14.0 (7.25-18.5)	5.0 (3.0-11.0)	0.0007
Day 14	11.5 (6.5-18.0)	4.0 (3.0-5.0)	<0.0001
D Day 7-0	-4.0 (-6.75 - +4.5)	-8.0 (-13.0 - -1.0)	0.017
ESR decrease of >10 mm/1h between Days 0 and 7	16.1%	41.4%	n/a
D Day 14-0	-4.5 (-11.0 - +4.25)	-11.5 (-19.25 - -2.75)	0.003
ESR decrease of >10 mm/1h between Days 0 and 14	30%	59.4%	n/a
LDH (U/L), median (95% CI)			
Pre-COVID	175 (158-211)	183 (156-199)	n/s (.76)
Day 0	207 (175-234)	200 (172-222)	n/s (.69)
Day 7	210 (179-249)	165 (144-198)	0.0013
Day 14	177 (154-202)	147 (135-160)	0.0004
D Day 7-0	+4 (-32.5 - +40.25)	-21 (-53 - +18)	0.087
LDH reduction of >30 U/L between Days 0 and 7	26.5%	50.0%	n/a
D Day 14-0	-26 (-68 - -10)	-35 (-76 - -15)	n/s (.31)
LDH reduction of >30 U/L between Days 0 and 14	46.4%	64.3%	n/a
Ultrassensitive troponin (ng/mL), median (IQR)			
Pre-COVID	n/a	n/a	n/a
Day 0	0.008 (0.005-0.012)	0.010 (0.008-0.015)	n/s (.23)
Day 7	0.007 (0.006-0.010)	0.005 (0.003-0.009)	0.048
Day 14	0.005 (0.004-0.008)	0.004 (0.003-0.005)	n/s (.14)
D Day 7-0	-0.003 (-0.005 - 0)	-0.004 (-0.007 - -0.003)	0.094
Troponin decrease of >0.003 ng/mL between Days 0 and 7	52.8%	80%	n/a
D Day 14-0	-0.003 (-0.007 - -0.001)	-0.007 (-0.011 - -0.004)	.16
Troponin decrease of >0.005 ng/mL between Days 0 and 7	33.3%	66.7%	n/a
D-dimer (ng/mL), median (95% CI)			
Pre-COVID	343 (215-427)	366 (255-438)	n/s (1.00)
Day 0	404 (289-575)	406 (266-584)	n/s (.77)
Day 7	310 (220-495)	189 (242-373)	n/s (.29)
Day 14	305 (216-420)	220 (200-306)	.019
D Day 7-0	-80 (-182 - +52)	-104 (-190 - -35)	n/s (.30)
D-dimer decrease between Days 0 and 7	63.6%	84.2%	n/a
D Day 14-0	-70 (-241 - +31)	-137 (-325 - -16)	n/s (.20)
D-dimer decrease of >100 ng/mL between Days 0 and 7	42.8%	61.6%	n/a
Ferritin (ng/mL), median (IQR)			
Pre-COVID	173 (133-241)	190 (113-310)	n/s (.61)
Day 0	389 (295-588)	471 (279-584)	n/s (.85)
Day 7	321 (248-476)	310 (198-476)	n/s (.41)
Day 14	240 (186-377)	241 (180-352)	n/s (.66)
D Day 7-0	-83 (-139 - -52)	-68 (-214 - -9)	n/s (.99)
D Day 14-0	-128 (-262 - -72)	-135 (-289 - -55)	n/s (.82)

On Day seven, 64.7% of men from the dutasteride group and 11.8% of men from the placebo group had undetectable nasopharyngeal SARS-CoV-2 virus or viral fragments (an increase of 444.9% in the percentage of subjects with virologic remission on Day seven when dutasteride was added; p=0.0094). On Day 14, 88.3% of the dutasteride group and 54.2% of the placebo group yielded non-detectable SARS-CoV-2.

On Day seven, the median usCRP was 0.34 mg/L in the dutasteride group and 1.47 mg/L in the placebo group (p<0.0001). On Day 14, the median usCRP was 0.36 in the dutasteride group and 0.39 in the placebo group (p=0.0026). Compared to baseline levels, usCRP was reduced on Day seven in 83.4% of men from the dutasteride group and 64.7% of men from the placebo group, and in 93.7% and 73.3% of men from dutasteride and placebo groups on Day 14, respectively.

The dutasteride group had lower lactate levels than the placebo group on Day seven (p=0.0049) and Day 14 (p=0.014). The goal of <0.5 mmol/L of increase between baseline (Day zero) and Day seven was achieved by 60% of subjects in the dutasteride group and by 19.2% of subjects in the placebo group (p=0.007).

Median ESR was 5.0 mm/1h in the dutasteride group and 14.0 mm/1h in the placebo group on Day seven (p=0.0007), and 4.0 mm/1h in the dutasteride group and 11.5 mm/1h in the placebo group (p<0.0001) on Day 14. Between Days zero and seven, ESR reduced by more than 10 mm/1h in 41.4% of men from the dutasteride group and in 16.1% from the placebo group. The goal of ESR reduction by more than 10 mm/1h from baseline (Day zero) to Day seven was achieved by 59.4% of men from the dutasteride group and 30.0% of those from the placebo group. LDH levels were statistically lower in the dutasteride group than the placebo group on Day seven (p=0.0013) and Day 14 (p=0.0004).

Ultrasensitive troponin levels were significantly lower on Day seven in the dutasteride group (median: 0.005 ng/mL) than in the placebo group (median: 0.007 ng/mL) (p=0.048). The goal of reduction of ultrasensitive troponin levels by more than 0.003 ng/mL between baseline (Day zero) and Day seven was achieved by 80% of participants in the dutasteride group and 52.8% in the placebo group. Ultrasensitive troponin levels reduced by more than 0.005 ng/mL in 66.7% of participants in the dutasteride group and 33.3% in the placebo group.

D-dimer level differences were only observed on Day 14, with a median D-dimer of 220 ng/mL in the dutasteride group and 305 ng/mL in the placebo group (p=0.019). Ferritin levels and changes between baseline (Day zero), Day seven, and Day 14 were similar between the dutasteride and placebo groups at all times and intervals.

**Figure 2:**
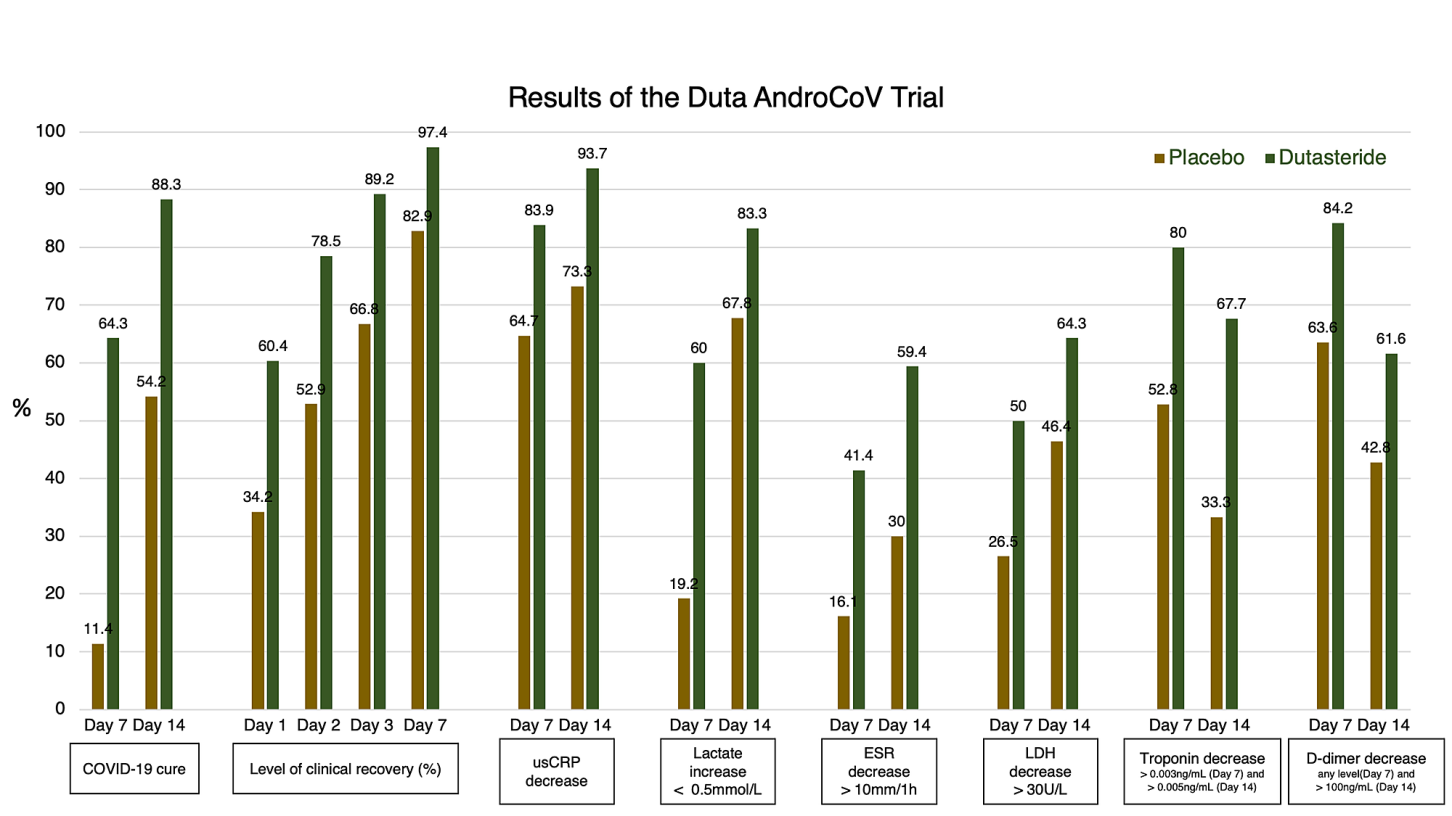
Main clinical and biochemical results of the EAT-DUTA AndroCoV-Trial EAT-DUTA AndroCoV: Early Antiandrogen Treatment With Dutasteride for COVID-19; usCRP: ultrasensitive C-reactive protein; ESR: erythrocyte sedimentation rate; LDH: lactate dehydrogenase

Of the 44 patients from the placebo group and 43 patients from the dutasteride group, 17 (38.6%) and 14 (32.5%) reported at least one adverse effect, except for changes in urine or sperm color or aspect, due to nitazoxanide use. All adverse effects fell under grade 1 and 2, and no serious adverse effects (SAEs) were reported; moreover, none of the patients had to interrupt treatment due to adverse effects. The most common adverse effects were diarrhea (12 and 11 subjects, respectively) and nausea (seven and six subjects, respectively). No hospitalizations, mechanical ventilation, or deaths were reported. Figure 2 summarizes the main clinical and biochemical findings of the present study.

## Discussion

Identifying effective treatments against COVID-19 remains a priority in the fight against the COVID-19 pandemic. To date, very few studies have been conducted to test interventions in the early stages of COVID-19 treatment. The majority of larger RCTs on pharmacological interventions for COVID-19 was conducted in later stages of the disease, when the timing for antiviral approaches was no longer appropriate. These limitations precluded us from conclusive findings on the efficacy of most drug regimens to date.

The virological and biochemical subset in the current study (n=87) was representative of the overall RCT subjects (n=130) since both demonstrated similar response patterns, including reductions between 40% and 50% in symptom duration and overall time to remission, differences in the recovery that were detected as early as on Day one, and two times faster recovery than the control group. After seven days, three times fewer men from the dutasteride group remained symptomatic than men from the placebo group. Accordingly, subjects from the dutasteride group demonstrated a faster recovery speed in terms of oxygen saturation.

Virological remission from COVID-19 can be determined by a negative RT-PCR-SARS-CoV-2, since, while detected, the virus may not necessarily reflect the presence of a viable and alive virus, a negative result after confirmation of COVID-19 is associated with almost 100% certainty for a cure. Approximately more than five times more males from the dutasteride group compared to those from the placebo group were cured of COVID-19 on Day seven, and four times more males from the placebo group maintained positive RT-PCR than males from the dutasteride group on Day 14. The substantial differences provide overwhelming evidence for the antiviral activity of dutasteride during early COVID-19.

The reductions observed in biochemical parameters related to inflammatory responses to SARS-CoV-2, including reductions in usCRP, lactate, ESR, and LDH levels in the dutasteride group, provide additional evidence of the protective role of dutasteride in the treatment of COVID-19. Although inflammation was not overtly detected in any of the groups, the arresting of the progression to inflammatory states may have contributed to the shortened disease duration. Indeed, biochemical findings support the patient-reported outcomes of improvements of symptoms and recovery rates in the dutasteride group.

Overall, differences were more substantial on Day seven than Day 14, which allows us to hypothesize that an important impact of dutasteride is to increase the speed of COVID-19 remission. Additional benefits included the reduction of the period of viral transmission once the treatment was initiated, with consequent earlier return to activities.

While androgens are both circulating and produced in tissue, elevated tissue DHT, implicated in AGA and benign prostatic hyperplasia, and prostate cancer, as well as AR sensitivity, may be better predictors of COVID-19 severity than circulating androgen levels. Changes in serum testosterone and DHT in response to dutasteride occur as early as one to two days after dutasteride initiation, which is shorter than the period of the present RCT [19]. In addition, in-cell and within-tissue concentration of these hormones occur earlier than the changes observed in the sera [20], eventually leading to a rapid reduction of TMPRSS-2 expression, which may help explain the efficacy of dutasteride observed in this study.

The present findings reinforce the previous results of our observational study (Pre-AndroCoV Trial) [21], which demonstrated that early antiandrogen therapy with dutasteride 0.5 mg per day or spironolactone 100 mg twice a day improved clinical and virologic outcomes in patients treated with azithromycin combined with either hydroxychloroquine, ivermectin, or nitazoxanide.

Whether dutasteride is effective against COVID-19 when used alone is uncertain although likely. When used chronically, evidence suggests a protective role of dutasteride in reducing COVID-19 disease severity. In a previous observational prospective study of COVID-19 among hospitalized men, we have observed that chronic users of dutasteride, despite being older and with more cardiac comorbidities, had reduced chances of being admitted to the intensive care unit [6]. The hypothesis of the combination of different drugs working synergistically follows the rationale of the complex SARS-CoV-2 pathogenicity and infectivity mechanisms of action.

The increased speed of recovery with the use of dutasteride may be particularly useful for patients between four and seven days after the initiation of symptoms, which corresponds to the later periods of the early stage of COVID-19, when the majority of the subjects seek medical assistance. When COVID-19 is detected during this period, therapeutic interventions must provide faster responses to avoid progression to inflammatory stages and acute lung injury.

Here we demonstrated in a randomized, double-blinded, placebo-controlled interventional study that men treated with dutasteride, a comprehensive and potent 5ARi, exhibited reduced viral load, clinical symptoms, disease course, and inflammatory responses induced by COVID-19 when compared to nitazoxanide and azithromycin alone. Whether similar results can be observed with lower doses, other 5ARis, or other antiandrogens is uncertain. Due to the lack of safety profile and uncertain efficacy for women, we do not recommend the use of dutasteride for this population until specific RCTs involving women are conducted.

Stronger antiandrogens, such as proxalutamide, a novel non-steroidal antiandrogen (NSAA), may provide further benefits in the treatment of COVID-19, and are being currently investigated in a double-blind, placebo-controlled RCT by our group.

## Conclusions

The findings of this randomized, double-blinded, placebo-controlled clinical trial suggest that that the addition of an early antiandrogen therapy with dutasteride to nitazoxanide and azithromycin for COVID-19 can effectively reduce SARS-CoV-2 viral shedding, improve clinical symptoms, and prevent inflammatory responses when compared to treatment with nitazoxanide plus azithromycin alone.
